# Heart failure and cardiogenic shock associated with the TB-immune reconstitution inflammatory syndrome

**DOI:** 10.5830/CVJA-2011-062

**Published:** 2012-04

**Authors:** CHRIS KENYON, NESHAAD SCHRUEDER, GRAEME MEINTJES, CHRIS KENYON, GRAEME MEINTJES, NESHAAD SCHRUEDER, MPIKO NTSEKHE, GRAEME MEINTJES, GRAEME MEINTJES

**Affiliations:** Department of Medicine, GF Jooste Hospital, Cape Town, South Africa; Department of Medicine, GF Jooste Hospital, Cape Town, South Africa; Department of Medicine, GF Jooste Hospital, Cape Town, South Africa; Division of Infectious Diseases and HIV Medicine, Department of Medicine, University of Cape Town, South Africa; Division of Infectious Diseases and HIV Medicine, Department of Medicine, University of Cape Town, South Africa; Department of Medicine, University of Cape Town, South Africa; Division of Cardiology, Department of Medicine, University of Cape Town, South Africa; Institute of Infectious Diseases and Molecular Medicine, University of Cape Town, South Africa; Imperial College London, UK

**Keywords:** TB-IRIS, heart failure, HIV, antiretroviral

## Abstract

Heart failure has not been described in the setting of TB-immune reconstitution inflammatory syndrome (IRIS). We describe a case of cardiogenic shock in the setting of TB-IRIS four weeks after commencement of antiretroviral therapy. Possible aetiologies and pathophysiology as well as suggested diagnostic and therapeutic approaches to this problem are discussed.

## Case report

A 34-year-old man was diagnosed with pulmonary tuberculosis (TB). *Mycobacterium tuberculosis* (susceptible to rifampicin and isoniazid) was cultured from his sputum. After commencing the intensive phase of TB treatment (rifampicin, isoniazid, pyrazinamide and ethambutol) his TB symptoms improved. He tested HIV seropositive and his CD_4_ count was 34 cells/ml. He commenced antiretroviral therapy (ART) with stavudine, lamivudine and efavirenz two weeks after starting TB treatment. Four days after ART initiation he developed a dry cough, worsening shortness of breath, central cramping abdominal pains, and recurrence of drenching night sweats.

He was admitted to another hospital and was treated with high-dose cotrimoxazole and prednisone 80 mg daily for a presumptive diagnosis of *Pneumocystis jiroveci* pneumonia (which in our opinion was an incorrect diagnosis). ART was temporarily interrupted then restarted. He initially responded to this therapy, but symptoms worsened five days after he had completed a 14-day course of prednisone. Over the next few days, he developed progressively worsening shortness of breath as well as orthopnoea, paroxysmal nocturnal dyspnoea and pedal oedema. He was then referred to our facility, four weeks after the initiation of ART.

On admission, he was in severe cardiogenic shock. His peripheries were cold and radial pulses were not palpable. His systolic blood pressure, detectable only on palpation, was 60 mmHg. He had a regular heart rate of 130 beats/min. His apex beat was laterally displaced, diffuse and hypokinetic, his jugular venous pressure was elevated 10 cm and there was a third heart sound. On chest auscultation there were extensive bilateral coarse crepitations. He had tender hepatomegaly.

**Fig. 1.**
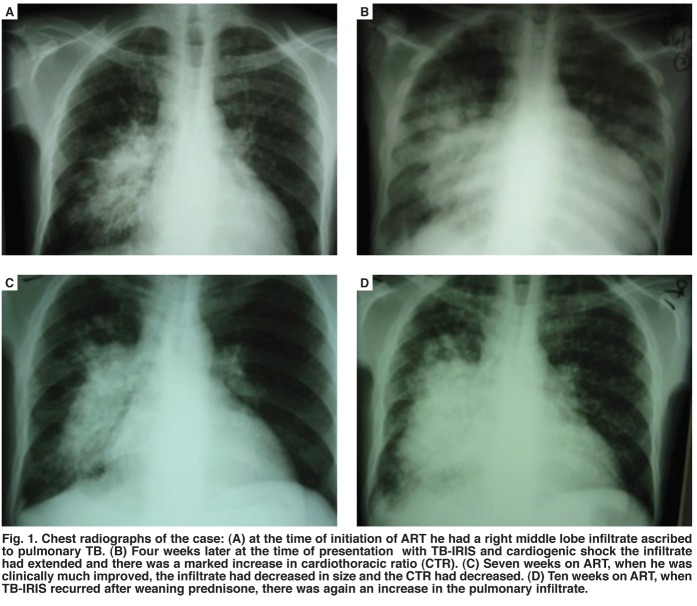
Chest radiographs of the case: (A) at the time of initiation of ART he had a right middle lobe infiltrate ascribed to pulmonary TB. (B) Four weeks later at the time of presentation with TB-IRIS and cardiogenic shock the infiltrate had extended and there was a marked increase in cardiothoracic ratio (CTR). (C) Seven weeks on ART, when he was clinically much improved, the infiltrate had decreased in size and the CTR had decreased. (D) Ten weeks on ART, when TB-IRIS recurred after weaning prednisone, there was again an increase in the pulmonary infiltrate.

**Fig. 2.**
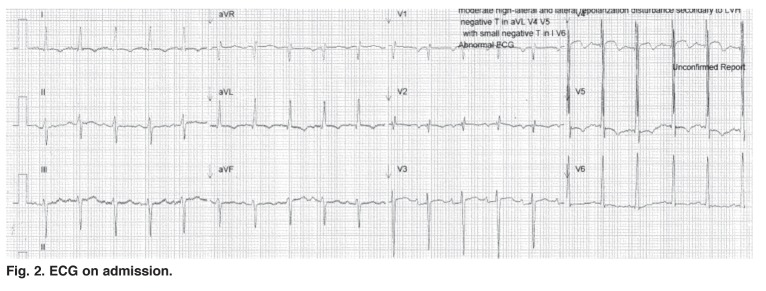
ECG on admission.

A chest radiograph showed worsening of the right mid-zone infiltrate that had been present prior to ART and was related to his pulmonary TB, as well as a marked increase in the cardiothoracic ratio (Fig. 1). Echocardiography confirmed a globally dilated heart with a fractional shortening of 13%. The ECG on presentation showed sinus tachycardia with a heart rate of 122 beats/min (Fig. 2). The QRS axis was –30 degrees, there was evidence of both left atrial and left ventricular hypertrophy by Cornell criteria,^1^ the QTc interval was prolonged at 453 ms, and there were widespread non-specific T-wave abnormalities. No other QRS or ST-segment abnormalities were noted.

A cardiac magnetic resonance image (MRI), performed after the patient was stabilised, showed no evidence of focal inflammation in the myocardium. The left ventricular ejection fraction was 15% on the MRI. Renal function was normal, haemoglobin was 10.6 g/dl, white cell count was 2.3 × 10^9^/l, platelet count was 163 × 10^9^/l and thyroid stimulating hormone level was 3.16 mU/l (normal range = 0.49–5.66). There was no history of significant ethanol abuse to suggest alcoholic cardiomyopathy.

Two diagnoses were made. Firstly, we made a diagnosis of paradoxical TB-associated immune reconstitution inflammatory syndrome (TB-IRIS). This was supported by his initial diagnosis of drug-susceptible pulmonary TB, his response to TB treatment prior to ART and then recurrence of TB symptoms associated with worsening of the pre-existing infiltrate of TB on the chest radiograph soon after ART commencement. Secondly, the patient had presented with severe acute heart failure four weeks after starting ART, coincident with the development of TB-IRIS. The cause of his heart failure was unclear and our considerations regarding the aetiology are discussed below.

He was transferred to the high-care unit and commenced on a dobutamine infusion, and once his blood pressure had improved over the next few days, furosemide, enalapril and spironolactone were introduced and the dobutamine weaned. His ART was stopped and he was recommenced on prednisone 100 mg daily initially. TB treatment was continued. Broad-spectrum antibiotics (ampicillin plus amikacin) were prescribed to cover the possibility of a bacterial pneumonia, but a blood culture was negative. He was also prescribed intravenous thiamine. Carvedilol was added later. ART was re-introduced after two weeks.

During the next two months, two attempts at weaning prednisone resulted in a return of his night sweats, dyspnoea, dry cough and worsening of his pre-existing infiltrates (Fig. 1) but not worsening of heart failure. On both occasions, he had rapid symptomatic improvement after reintroduction of high-dose prednisone. At evaluation three months after initial presentation, he was able to walk two kilometres on the flat, had no signs of heart failure and a repeat echocardiogram measured his fractional shortening as 22%. Prednisone was gradually weaned and he received six months in total. After six months on ART his CD_4_ count was 359 cells/ml, his HIV viral load was undetectable and his sputum TB culture was negative.

## Discussion

## Paradoxical TB-IRIS

TB-IRIS affects approximately eight to 43% of patients who commence ART while on treatment for active TB, and it manifests with recurrence or new symptoms, clinical signs and/ or radiographic features of TB despite effective TB treatment.^2^ A recent meta-analysis reported the pooled cumulative incidence across studies as 15.7%.^3^ Onset is typically within the first four weeks of ART. IRIS is thought to result from rapid but dysregulated restoration of antigen-specific immunity during early ART.^4^ In paradoxical TB-IRIS this results in inflammation at the sites of TB disease directed at the residual MTB antigen.^5^ In addition, TB-IRIS has been shown to be accompanied by marked cytokine elevation in peripheral blood, in particular the pro-inflammatory cytokines IL-6, TNF-α and IFN-γ.^6^

Since high levels of these pro-inflammatory cytokines have been implicated in the pathogenesis of HIV-associated dilated cardiomyopathy (DCMO),^6^ it would be plausible to expect that patients who experience severe TB-IRIS accompanied by high pro-inflammatory cytokine concentrations and have pre-existing subclinical DCMO could develop overt heart failure as a consequence. Both TNF-α and inducible nitric oxide synthetase (the production of which is increased in the presence of TNF-α and IL-6) have been implicated in the pathogenesis of DCMO in patients with and without HIV infection.^6^ It is thought that by a similar cytokine-mediated mechanism, bacterial sepsis may precipitate myocardial depression, particularly in patients with underlying cardiac disease.^7^ The cytokine-mediated upregulation of inducible nitric oxide synthetase in sepsis results in increased production of reactive oxygen species and interference with calcium homeostasis, thereby impairing myocyte contractility.^8^

## HIV cardiomyopathy

DCMO is common in late-stage HIV infection. A Rwandan study found the prevalence of echocardiogram-diagnosed DCMO to be 17.7% among 416 consecutively screened HIV-infected patients without a previous history of heart disease.^9^ In autopsy studies, myocarditis is present in nine to 52% of HIV-infected patients. In only 10 to 15% of these cases of myocarditis can evidence of a bacterial, fungal or protozoal infection be found.^10^-^12^ The majority of other cases of DCMO are thought to be a result of the direct or immune-mediated effects of HIV itself.

A direct role for HIV infection in the development of DCMO was suggested by a study of 952 asymptomatic HIV-infected patients who were followed for 60 months.^13^ DCMO was diagnosed by echocardiogram in 8%, most of whom had evidence of a myocarditis on endomyocardial biopsy. Among these patients, 76% had HIV nucleic acid sequences present in the myocytes, while 26% had evidence of Coxsackie virus, cytomegalovirus, or Epstein-Barr virus infection.

HIV may induce myocardial injury through three putative mechanisms.^14^ Firstly, HIV may have a direct cytotoxic effect on the myocytes.^15^ Secondly, HIV-related immune activation may result in high levels of pro-inflammatory cytokines, which result in dysfunction and apoptosis of cardiac myocytes. Thirdly, this immune activation may drive an auto-immune reaction targeting host myocardial cells.

## IRIS myocarditis

IRIS may affect any organ system and has been described in association with a wide variety of pathogens as well as autoimmune conditions and with HIV itself.^3^ To our knowledge, there has been one published case of IRIS-associated myocarditis. The patient was a 29-year-old HIV-infected man with a CD_4_ count of 56 cells/ml who presented five weeks after commencing ART, with acute myocarditis complicated by fatal polymorphic ventricular tachycardia.^16^

At post mortem, he was found to have evidence of a cytomegalovirus (CMV) pneumonia and a predominantly CD_8_ lymphocytic infiltrate of his myocardium and conduction system. Immunohistochemical staining for CMV in his myocardium was negative. The myocarditis was attributed to IRIS, given the timing of the presentation, the marked reduction in HIV viral load on ART and the prominent inflammatory infiltrate of the myocardium. The antigenic target of the IRIS was likely HIV itself or an undiagnosed pathogen.

In the case we present here, it was not possible to exclude a cellular infiltrate of the myocardium due to IRIS myocarditis because we did not perform an endomyocardial biopsy, but the MRI findings did not suggest myocardial inflammation, making this unlikely.^17^ We also considered the possibility that our patient’s heart failure resulted from inflammation in the myocardium due to TB-IRIS myocarditis. However, because of the MRI showing no evidence of inflammation in the myocardium, we think this unlikely. We would expect TB myocarditis to be characterised by areas of focal gadolinium enhancement on cardiac MRI.^18^

## Conclusion

This patient had no symptoms of heart failure prior to ART. After starting ART he developed paradoxical TB-IRIS, a diagnosis that we made on the basis of the characteristic clinical features described above and which was supported by his having exacerbations of TB-IRIS symptoms on weaning prednisone but improvement when recommenced on higher doses. Prednisone has been shown to result in symptomatic improvement in TB-IRIS.^19^

On admission to our hospital, the patient had features of acute, severe heart failure; the heart shadow had increased markedly in size on chest radiograph and he was in cardiogenic shock. He had a good response to inotropes, anti-failure treatment, high-dose prednisone and temporary interruption of ART. There was no cause other than HIV found for his DCMO. We hypothesise that he had an underlying subclinical HIV-associated DCMO, and the development of TB-IRIS with the associated hypercytokinaemia resulted in rapid and severe deterioration in cardiac function.

The precipitation of heart failure during IRIS seems to be a rare event. One explanation for this is that the diagnosis may be missed. We would therefore encourage clinicians to at least entertain the diagnosis in patients who present with features of heart failure soon after starting ART, and especially if they present with other features of IRIS. The optimal management of such patients is not determined and will depend on the severity and co-morbidities. In severe cases, consideration should be given to ART interruption and corticosteroid therapy in addition to anti-failure therapy. Given the rarity of this event, however, there is no prospective evidence on which to base management decisions. Prospective studies of changes in cardiac function using echocardiography in patients starting on ART are warranted. So too are autopsy studies that include examination of the myocardium of patients who die during periods of IRIS.
